# The Influence of Vascular Function on Biomarkers for Alzheimer's Disease in Adults with Chronic Cerebral Hypoperfusion

**DOI:** 10.1002/alz70856_106791

**Published:** 2026-01-08

**Authors:** Brandon G. Fico, M. Erin Moir, Sarean Harmoni A. Gaynor‐Metzinger, Nicole A. Loggie, Alexander M. Norby, Anna J. Howery, Alexandre Hamaide, Leonardo A. Rivera‐Rivera, Oliver Wieben, Kevin M. Johnson, Sterling C Johnson, Jill N. Barnes

**Affiliations:** ^1^ University of Wisconsin‐Madison, Madison, WI, USA; ^2^ Florida Atlantic University, Boca Raton, FL, USA; ^3^ Wisconsin Alzheimer's Disease Research Center, University of Wisconsin School of Medicine and Public Health, Madison, WI, USA

## Abstract

**Background:**

Cerebral hypoperfusion can result in cognitive decline and is a risk factor for Alzheimer's disease (AD). A common cerebral anatomical variation (vertebral artery hypoplasia [VAH]) has been linked to reduced cerebral blood flow and elevated cerebral pulsatility. Additionally, increased blood pressure (BP) and arterial stiffness are associated with elevated cerebral pulsatility and may increase risk for neurodegeneration and AD in those with VAH. Therefore, the purpose of this study was to determine the influence of BP and arterial stiffness on cerebral pulsatility and biomarkers for neurodegeneration and AD in adults with and without VAH.

**Methods:**

Sixty‐five cognitively unimpaired healthy older adults (64±4 years; 52 females) with VAH (*n* = 17) and without VAH (noVAH; *n* = 48) were included in the study. Brachial BP, arterial stiffness (carotid‐femoral pulse wave velocity), serum blood samples, and 3T magnetic resonance imaging (MRI) were assessed. Serum samples were analyzed for neurofilament light chain (NfL) and phospho‐tau 217 (pTau 217). Intracranial pulsatility index (PI) was quantified using 4D flow MRI in the internal carotid arteries (ICAs), anterior cerebral arteries (ACAs), middle cerebral arteries (MCAs), vertebral arteries (VAs), and basilar artery (BA).

**Results:**

There were no group differences in BP, arterial stiffness, or cerebral PI (in the ICAs, MCAs, VAs, and BA) between VAH and noVAH (*p* >0.05 for all). However, the VAH group had greater ACAs PI (*p* <0.01) compared with the noVAH group. Systolic BP was positively associated with cerebral PI in the MCAs in the VAH group only (r=0.63, *p* <0.01), as there was no association in the noVAH group (r=0.08, *p* = 0.60). There were no differences in serum NfL or pTau 217 biomarkers between VAH and noVAH groups (*p* >0.05 for both). In addition, systolic BP was inversely associated with pTau 217 (r=‐0.80, *p* <0.01) in the VAH group only, as there was no association in the noVAH group (r=‐0.04, *p* >0.83).

**Conclusions:**

Adults with VAH had greater cerebral pulsatility in the anterior circulation compared with adults without VAH. In adults with VAH, increased systolic BP resulted in elevated cerebral pulsatility and potentially acting as a compensatory mechanism for decreased perfusion pressure, the increased systolic BP resulted in a reduction in pTau 217 levels.

